# Bait attractiveness changes community metrics in dung beetles (Coleoptera: Scarabaeidae: Scarabaeinae)

**DOI:** 10.1002/ece3.9975

**Published:** 2023-04-07

**Authors:** Andressa Bach, Lúcia A. F. Mateus, Carlos A. Peres, Torbjørn Haugaasen, Julio Louzada, Joseph E. Hawes, Renato A. Azevedo, Emanuelly F. Lucena, José Victor A. Ferreira, Fernando Z. Vaz‐de‐Mello

**Affiliations:** ^1^ Programa de Pós‐Graduação em Ecologia e Conservação da Biodiversidade, Laboratório de Scarabaeoidologia, Instituto de Biociências Universidade Federal de Mato Grosso Avenida Fernando Corrêa da Costa, n° 2367, Boa Esperança 78060900 Cuiabá Brazil; ^2^ Programa de Pós‐Graduação em Ecologia e Conservação da Biodiversidade, Laboratório de Ecologia e Manejo de Recursos Pesqueiros, Departamento de Botânica e Ecologia, Instituto de Biociências Universidade Federal de Mato Grosso Avenida Fernando Corrêa da Costa, n° 2367, Boa Esperança 78060900 Cuiabá Brazil; ^3^ School of Environmental Sciences University of East Anglia Norwich NR4 7TJ UK; ^4^ Faculty of Environmental Sciences and Natural Resource Management Norwegian University of Life Sciences Universitetstunet 3 1430 Ås Norway; ^5^ Departamento de Ecologia e Conservação Universidade Federal de Lavras 37203202 Lavras Brazil; ^6^ Coordenação de Biodiversidade‐COBIO, Instituto Nacional de Pesquisas da Amazônia – INPA Av. André Araújo, 2936, Petrópolis 69083‐000 Manaus Brazil; ^7^ Laboratório de Termitologia, Departamento de Sistemática e Ecologia, Centro de Ciências Exatas e da Natureza Universidade Federal da Paraíba Campus I Lot. Cidade Universitária 58051‐900 João Pessoa Brazil; ^8^ Programa de Pós‐Graduação em Ecologia e Conservação da Biodiversidade Universidade Estadual de Santa Cruz Rodovia Jorge Amado, Km 16, 45662900, Salobrinho Ilhéus Brazil

**Keywords:** Brazilian Amazon, community structure, flight interception trap, primary forest, *terra firme*

## Abstract

Species relative abundance (SRA) is an essential attribute of biotic communities, which can provide an accurate description of community structure. However, the sampling method used may have a direct influence on SRA quantification, since the use of attractants (e.g., baits, light, and pheromones) can introduce additional sources of variation in trap performance. We tested how sampling aided by baits affect community data and therefore alter derived metrics. We tested our hypothesis on dung beetles using data from flight interception traps (FITs) as a baseline to evaluate baited pitfall trap performance. Our objective was to assess the effect of bait attractiveness on estimates of SRA and assemblage metrics when sampled by pitfall traps baited with human feces.Dung beetles were sampled at three *terra firme* primary forest sites in the Brazilian Amazon. To achieve our objective, we (i) identified species with variable levels of attraction to pitfall baited with human feces; (ii) assessed differences in SRA; and (iii) assessed the effect of bait on the most commonly used diversity metrics derived from relative abundance (Shannon and Simpson indices). We identified species less and highly attracted to the baits used, because most attracted species showed greater relative abundances within baited pitfall traps samples compared with our baseline. Assemblages sampled by baited pitfall traps tend to show lower diversity and higher dominance than those sampled by unbaited FITs. Our findings suggest that for ecological questions focused on species relative abundance, baited pitfall traps may lead to inaccurate conclusions regarding assemblage structure. Although tested on dung beetles, we suggest that the same effect could be observed for other insect taxa that are also sampled with baited traps. We highlight a need for further studies on other groups to elucidate any potential effects of using baits.

## Graphical abstract

Assemblages sampled by baited pitfall traps tend to show lower diversity and higher dominance than those sampled by unbaited flight interception traps. Therefore, for ecological questions focused on species relative abundance, baited pitfall traps may lead to inaccurate conclusions regarding assemblage structure.

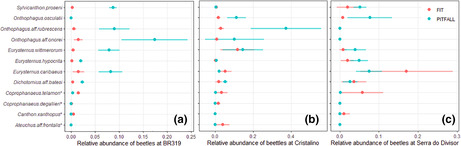

## 1 INTRODUCTION

The study of communities allows ecologists to draw inferences about biodiversity (Magurran, 1991), which requires estimates of community attributes (Begon et al., 2006). Although attributes may use data on taxonomic composition (e.g., presence/absence of taxa), considering species abundance patterns provide more detailed description of the community (Peroni & Hernández, 2011). Species relative abundance (SRA) is an essential property of community structure (Holt, 1997), and studies of species commonness or rarity may lead to a better understanding of communities (Anderson et al., 2011). Nevertheless, the choice of sampling method has a direct influence on the community quantification (Campos et al., 2000) because the effectiveness of each sampling method varies among taxa (Katsanevakis et al., 2012; Missa et al., 2009).

Traps, which are widely used to collect a wide range of insects (Juillet, 1963), can be broken into two types: those that capture individuals randomly and those that use some kind of lure to attract insects into the trap (Henderson & Southwood, 2016). For example, flight interception traps (FITs) are a passive method (Matthews & Matthews, 1972) used to collect active flying insects (Campos et al., 2000; Lamarre et al., 2012; Peck & Davies, 1980), providing a random sample of individuals that move through trap height (Ozanne, 2005). In comparison, pitfall traps are a standard method to capture ground‐active insects (Southwood, 1978; Ward et al., 2001) and may incorporate baits to attract insects with any given food preference (Almeida et al., 1998; Woodcock, 2005). Dung beetles (Coleoptera: Scarabaeidae: Scarabaeinae) are a high‐performance indicator group (Gardner et al., 2008), highly suitable for biodiversity monitoring and assessments in tropical forests (Favila & Halffter, 1997; Halffter & Favila, 1993; Lobo et al., 1988). They primarily consume mammal dung (Gill, 1991), but may also feed on carcasses, decaying plant material, and fungi (Bornemissza, 1971; Halffter & Matthews, 1966). Dung beetles are commonly sampled using baited pitfall traps (Doube & Giller, 1990; Raine & Slade, 2019; Silva et al., 2012), whereas FITs are much less commonly used (Da Costa et al., 2009; Puker et al., 2020; Touroult et al., 2017).

The capture effectiveness of pitfall traps for dung beetles has been tested with a wide range of baits including feces from different mammal species (Estrada et al., 1993; Ferreira et al., 2020) or a combination of feces in various proportions (Marsh et al., 2013), as well as decaying meat, and fruits (Beiroz et al., 2016; Silva et al., 2007). These studies show that different dung beetle species are attracted to different bait types (Filgueiras et al., 2009; Silva et al., 2012; Tsuji et al., 2021). It appears that dung beetles are more attracted to feces of omnivorous mammals (Whipple & Hoback, 2012), especially human feces at least in the Neotropical region (Milhomem et al., 2003). The expected species pool represented by pitfall traps baited with human feces is comprised of coprophagous or generalist species, thereby excluding other species with divergent feeding habits. In contrast, FITs may provide a broader inventory of the dung beetle species (Davis et al., 2001), even though the expected species pool for FITs only includes taxa that typically fly at the trap height and excludes flightless species. FITs seem to capture fewer dung beetle individuals and species overall, compared with baited pitfall traps (Audino et al., 2011; Da Silva et al., 2011). However, many studies use a large number of pitfall traps, but allocate limited time and spatial replication to FITs, and thereby hindering comparability in the relative sampling effort between the two methods.

Due to the widespread application of baited pitfall traps in biodiversity studies worldwide, it is essential to quantify possible sampling biases that may affect their performance and limit our interpretation of results from dung beetles surveys. Bait quality (Álvarez et al., 2021; Souza et al., 2015), desiccation resistance (Lucci Freitas et al., 2014; Newton & Peck, 1975), trap size (LeBlanc et al., 2021), and even the position within the pitfall traps can all influence attraction (Lobo et al., 1988). Beyond these factors, there are also idiosyncratic species responses due to food preferences (Almeida et al., 1998; Larsen et al., 2006; Noriega, 2012). The use of baits may therefore result in incomplete or misrepresented information on species abundance patterns, thereby affecting estimates of community structure.

Here, to explore the effects of baited traps on community metrics, we used the dung beetle (Coleoptera: Scarabaeidae: Scarabaeinae) fauna of the western Brazilian Amazon as a model group, and compared the community composition between unbaited FITs and pitfall traps baited with human feces using the former as a baseline. There is no knowledge of flightless dung beetles species in our study area (F. Z. Vaz‐de‐Mello, pers. obs.), and we therefore assume that the expected species pool from pitfall traps is nested within the expected species pool from FITs. We hypothesize that bait affects community data, and therefore alter community metrics. Specifically, we predict that the over‐representation of the most attracted species will alter community metrics, resulting in increased dominance. To test our hypothesis, we aimed to (i) identify species with variable levels of attraction to pitfall traps baited with human feces; (ii) assess differences in SRA between baited pitfalls and unbaited FITs; and (iii) assess the effect of baited traps on dung beetle assemblage metrics.

## 2 MATERIALS AND METHODS

### 2.1 Study area

**FIGURE 1:**
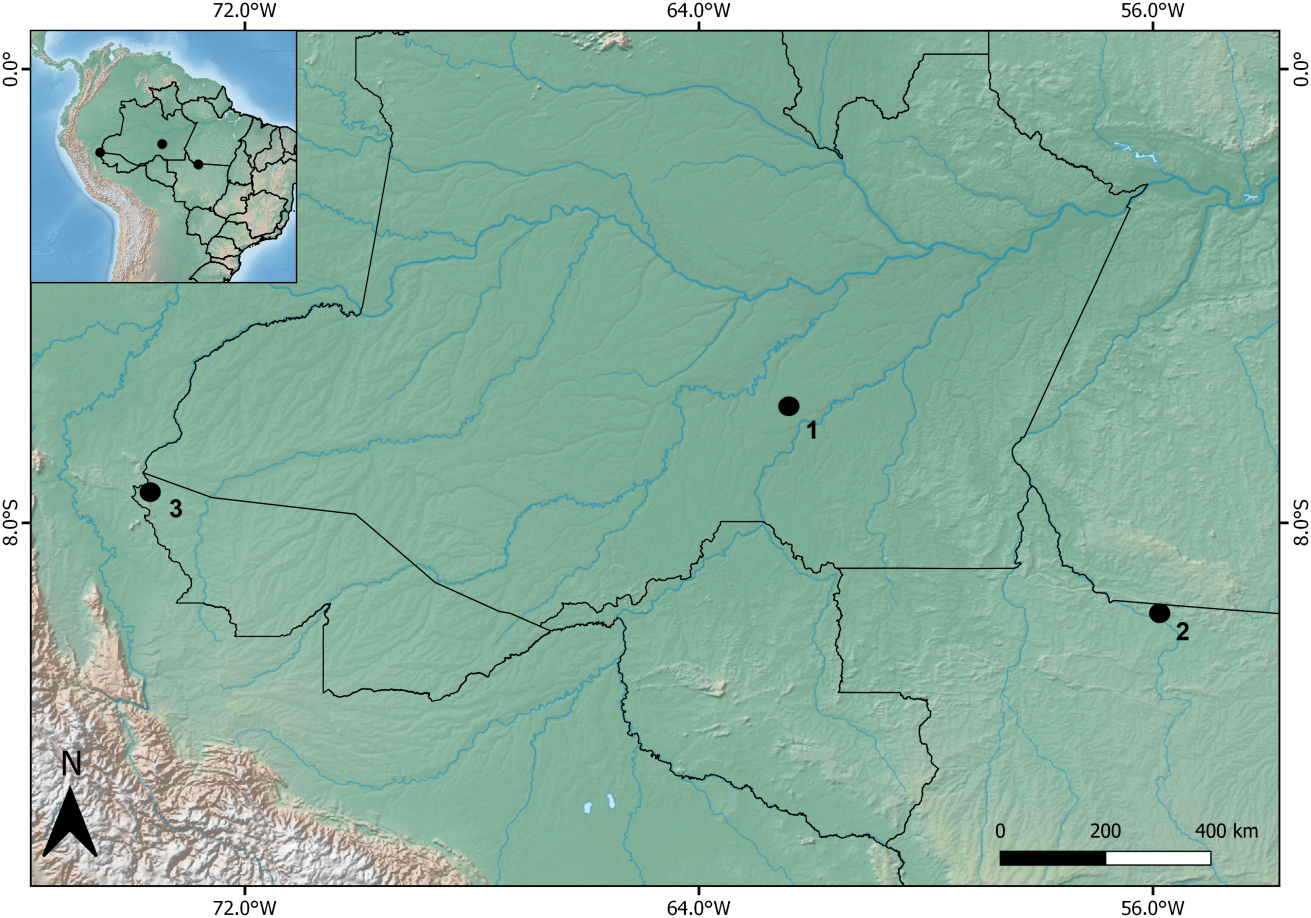
Study region map showing the location of three primary forest areas of terra firme within the Brazilian Amazon (1. BR‐319; 2. Cristalino; and 3. Serra do Divisor) where dung beetles were sampled with flight interception, and baited pitfall traps. Map created in QGIS version 3.8.2.

Dung beetles were sampled from October to December 2019 at three localities (Table S1) of lowland terra firme forest—that is, forest areas situated above the flood levels of rivers, streams, and lakes, in the Brazilian Amazon. We sampled at (i) the region of Lago Capanã Grande Extractive Reserve, Amazonas state (hereafter, BR‐319); (ii) the region of Cristalino State Park, Mato Grosso state (hereafter, Cristalino); and (iii) Serra do Divisor National Park, Acre state (hereafter, Serra do Divisor) (Figure 1). At each locality, dung beetles were sampled using FITs, and pitfall traps baited with human feces along three transects (Table S1) of 1000 m.

### 2.2 Sampling

Four FITs were placed every 250 m along each transect. These were open for 12 days, and checked every 96 h (4 days). Ten pitfall traps were placed every 100 m (Da Silva & Hernández, 2015), with 48 hours of trap exposure in the field (Figure S1). We considered every transect sampled by each method as a sampling unit. Hence, there are nine sampling units for FITs, and nine sampling units for pitfall traps. We assumed spatial independence between all transects, as the minimum distance was 1 km between them (although the mean straight‐line distance between transects is approx. 8 km). There are no temporal differences since pitfalls and FITs were operated simultaneously (Figure S1).

As FITs and pitfall traps were placed at least 50 m apart as suggested by Larsen and Forsyth (2005), we assume that is unlikely that FITs captures were affected by pitfall bait. We also assume that the available dung beetle assemblage was the same for both methods because FITs and pitfall traps were operated simultaneously, and dung beetles are estimated to travel only approximately 90 m during 48 h (at least in the Brazilian Atlantic Forest according to Da Silva & Hernández, 2015). We therefore assume that FITs and pitfall traps within the same transect will sample from the same assemblage, although all traps were spatially independent.

Here, we use FIT data as a baseline to assess the effect of baits on the measure of dung beetle SRA. Although unbaited pitfall traps may seem the obvious baseline to baited pitfall traps, this method captures few individuals (Chong & Hinson, 2015; Frizzas et al., 2020) because dung beetles disperse mainly by flight (Halffter & Edmonds, 1982) and are captured by baited pitfall traps essentially because they are attracted. Furthermore, most of dung beetles appear to fly below two meters in height (Lähteenmäki et al., 2015), and there is no knowledge of flightless species occurring at our three study areas in the Brazilian Amazon (F. Z. Vaz‐de‐Mello, pers. obs.). We assume therefore that the expected species pool for pitfall sampling is nested within the expected species pool for FITs. To support this assumption, we performed a nonmetric multidimensional scaling (NMDS) (“vegan” package; Oksanen et al., 2020) of all dung beetles SRA excluding singletons, and the results showed the expected nesting (Figure S2). As a result, we consider that FITs data provide a feasible baseline for dung beetles, although we are aware that there are other variables influencing both capture for both method (Table S2).

### 2.3 Identification

Dung beetle species were identified using identification keys, entomological collection for comparison, and taxonomic bibliography (Carvalho De Santana et al., 2019; Cook, 1998, 2000; Cupello & Vaz‐de‐Mello, 2018; Edmonds, 1994; Edmonds & Zídek, 2004, 2010, 2012; Génier, 1996, 2009; Génier & Arnaud, 2016; Rossini & Vaz‐de‐Mello, 2017; Rossini & Vaz‐de‐Mello, 2020; Rossini, Vaz‐de‐Mello, & Zunino, 2018; Rossini, Vaz‐de‐Mello, & Zunino, 2018; Silva & Valois, 2019; Vaz‐de‐Mello et al., 2011). All specimens were deposited at *Coleção Entomológica de Mato Grosso Eurides Furtado* (CEMT), at *Universidade Federal de Mato Grosso*, Cuiabá, Mato Grosso, Brazil.

### 2.4 Data analysis

To compare the sampling effort of FITs and pitfall traps, we used species accumulation curves based on the number of individuals using the vegan package (Oksanen et al., 2020). To assess the effect of bait on the accumulation curves, we only included species captured by baited pitfall traps (Table S3), assuming that these are attracted to bait.

Hotelling's *T* ^2^ test was performed to compare the assemblages sampled by FITs and pitfall traps. We used SRA as the response variable and trap (FITs or pitfall traps) as a predictor variable (“Hotelling” package; Curran, 2018). Singletons were excluded from this analysis (Table S3), as the capture of a single individual did not meet our objective. In total, 168 species were considered for Hotelling's *T* ^2^.

The indicator value (IndVal) was calculated to identify species associated with FITs or pitfall traps (“indicspecies” package, with 999 permutations; De Cáceres, 2020; De Cáceres & Legendre, 2009). IndVal is the product of two components (specificity and fidelity) multiplied by 100, to yield percentages. These components are calculated based on species abundance and occurrence (Dufrene & Legendre, 1997). Species were categorized as highly (*p* < .05 and IndVal ≥ 70%), moderately (*p* < .05, 45% ≤ IndVal < 70%), or weakly associated (when *p* < .05, IndVal < 45%) to each trapping method (as used by Tonelli et al., 2019; Verdú et al., 2011). Species highly associated were captured almost exclusively by one trap type with great abundance. Species moderately associated were capture exclusively or presented greater abundance in one trap type compared with the other. Species weakly associated were not captured exclusively to one trap type and had low abundance.

Not all dung beetle species are widely distributed across the Brazilian Amazon. To avoid the possibility that association strength between species and traps was altered because of a species not occurring in a particular location, IndVal was calculated for species sampled at all sites and species common to two sites using a pairwise comparison. (Table S3). We also evaluated the correlation between species and trap type using the Point–Biserial Correlation Coefficient. This division is even more relevant to the correlation coefficient, as the absence of species in samples with one type of trap increases the association strength as much as the presence of species in samples with the other trap (De Cáceres & Legendre, 2009).

To test whether the use of bait affects SRA between baited and the unbaited baseline, we used a chi‐squared goodness‐of‐fit test (“chisq.test” function), with standardized residuals (SR) as a post hoc method. Species that contributed to significance were those with *p* < .05 and SR outside of the range −1.96 to 1.96 (Callegari‐Jacques, 2003). Assuming that unbaited FITs samples reflect dung beetle assemblage structure better than baited pitfall traps, SRA sampled by FITs was used as the expected frequency, while SRA sampled by pitfall traps was used as the observed frequency. We selected species that were present in at least two sites and sampled by both FITs and pitfall traps (Table S3), and chi‐squared was always applied within the same transect.

We evaluated how baited and unbaited traps influences two indices based on SRA: Shannon's entropy index and the inverse of Simpson's concentration index, both used as measures of diversity and based on the Hill Numbers (Chao et al., 2014). We used the “iNEXT” package (Hsieh et al., 2022) for this analysis. Each index was calculated based on the assemblages captured by FITs and pitfall traps separately and the result was compared within the same transect. For these comparisons, we selected only species captured by pitfall traps (Table S3), assuming that these were attracted to the bait. Even if some individuals were not attracted to the bait and yet fell into the pitfall traps as a random event, this is likely to be a rare occurrence and comprise a low number of individuals. These will therefore likely have negligible influence on the overall result.

Analyses were performed on transect‐level data whenever possible, using R version 3.6.2 (R Core Team, 2019).

## 3 RESULTS

In total, 23,427 dung beetle individuals were sampled belonging to 198 species (Table S3), of which 55 species (27.78%) were captured exclusively by FITs, 35 species (17.68%) were captured exclusively by pitfall traps, and 108 species (54.54%) were collected by both.

Species accumulation curves were elaborated based on attracted species and showed that richness was similar between FITs and baited pitfall, as most confidence intervals were overlapping. Relative abundance differed significantly between FITs and pitfall trap samples (*T* ^2^ = 32.07; df: 27/168; *p* < .001). Five species were weakly associated with FITs (Table 1), and none were highly or moderately associated with this trap type. The same species were all negatively correlated with pitfall traps (*p* ≤ .016; Table S6). Eight species were highly associated with pitfall traps, while four were moderately associated, and 13 were weakly associated with this trap type. Pitfall‐associated species were positively correlated with pitfall traps (Table S6).

**TABLE 1:** IndVal and *p*‐value of dung beetle species highly, moderately, and weakly associated with unbaited flight interception traps and pitfall traps baited with human feces at three *terra firme* primary forest sites in the Brazilian Amazon.

Association strength	Species	IndVal (%)	*p*
Weakly associated with FITs	*Ateuchus* aff. *frontalis* (Boucomont, 1928)	30.12	.001
	*Coprophanaeus telamon* (Erichson, 1847)	27.08	.035
	*Coprophanaeus degallieri* (Arnaud, 1997)	20.62	.001
	*Canthon xanthopus* (Blanchard, 1846)	11.80	.017
	*Dendropaemon angustipennis* (Harold, 1869)	11.11	.011
Highly associated with pitfall	*Onthophagus* aff. *rubrescens* (Blanchard, 1846)	97.04	.001
	*Onthophagus osculatii* (Guérin‐Méneville, 1855)	94.95	.001
	*Onthophagus* aff. *onorei* (Zunino & Halffter, 1997)	89.23	.001
	*Eurysternus caribaeus* (Herbst, 1789)	79.6	.001
	*Eurysternus hypocrita* (Balthasar, 1939)	77.67	.001
	*Eurysternus wittmerorum* (Martinez, 1988)	76.03	.001
	*Dichotomius* aff. *batesi* (Harold, 1867)	73.4	.001
	*Sylvicanthon proseni* (Martínez, 1949)	71.72	.001
Moderately associated with pitfall	*Canthon luteicollis* (Erichson, 1847)	67.21	.001
	*Onthophagus onorei* (Zunino & Halffter, 1997)	65.00	.001
	*Onthophagus* aff. *osculatii* (Guérin‐Méneville, 1855)	59.83	.001
	*Eurysternus cayennensis* (Castelnau, 1840)	51.93	.002
Weakly associated with pitfall	*Eurysternus arnaudi* (Génier, 2009)	43.81	.001
	*Oxysternon conspicillatum* (Weber, 1801)	41.23	.001
	*Eurysternus hamaticollis* (Balthasar, 1939)	34.83	.001
	*Eurysternus strigilatus* (Génier, 2009)	28.53	.038
	*Deltochilum orbiculare* (Lansberge, 1874)	27.36	.001
	*Dichotomius mamillatus* (Felsche, 1901)	25.37	.001
	*Eurysternus foedus* (Guerin‐Meneville, 1844)	20.25	.001
	*Eurysternus ventricosus* (Gill, 1990)	18.33	.001
	*Canthon rufocoeruleus* (Martínez, 1948)	11.66	.003
	*Dichotomius robustus* (Luederwaldt 1935)	11.64	.001
	*Onthophagus digitifer* (Boucomont, 1932)	10.00	.011
	*Dichotomius melzeri* (Luederwaldt, 1922)	09.66	.018

**FIGURE 2:**
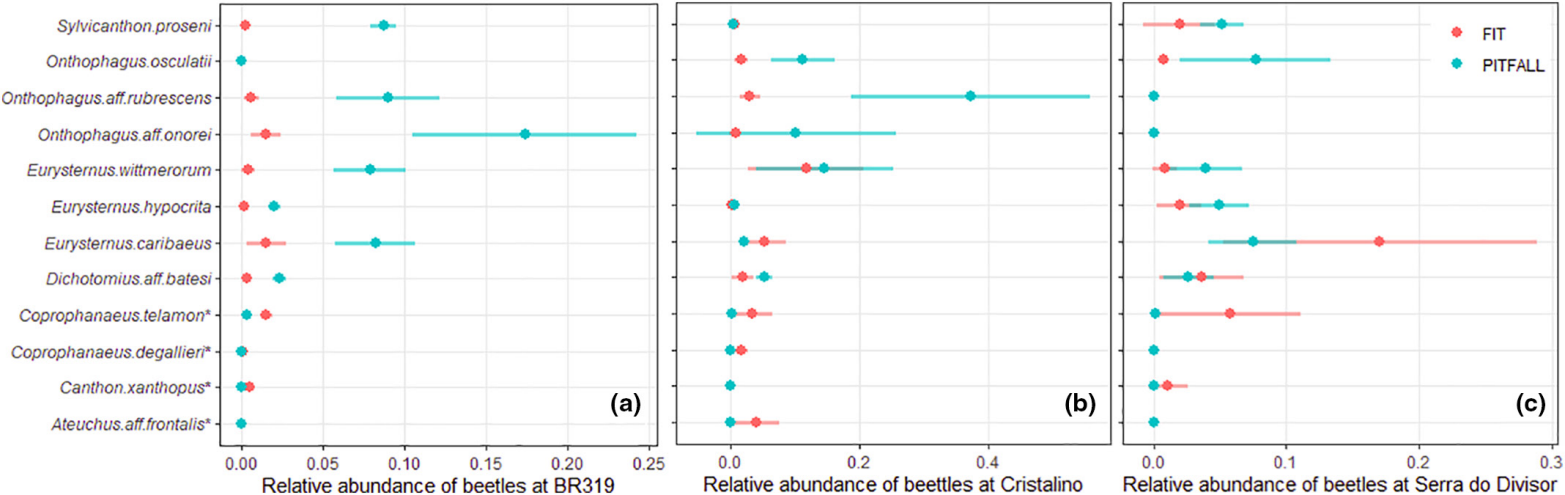
A comparison of the relative abundance of dung beetle species highly associated with pitfall traps and weakly associated with flight interceptions traps (FITs). The comparisons (chi‐squared goodness of fit) were made at transect level to show how species' relative abundance (SRA; mean ± standard deviation) changes when bait is used at three sites (a) BR‐319, Amazonas; (b) Cristalino; (c) Serra do Divisor. Graphics created with “tidyverse” and “ggtext” packages (Wickham et al., 2019; Wilke & Wiernik, 2022). *species associated with fligh interception traps.

Species relative abundance sampled by FITs and pitfall traps differed significantly (Table S5). FITs‐associated species showed a lower relative abundance in pitfall samples for all localities (Figure 2). Four species highly associated with pitfall traps—*E. hypocrita*, *O*. aff. *onorei*, *O*. aff. *rubrescens*, and *O. osculatii*—presented higher relative abundance in all pitfall trap samples (Figure 2). This pattern was not consistent for other species highly associated with pitfall traps, as in the case of *D*. aff. *batesi*, *E. caribaeus*, *E. wittmerorum*, and *S. proseni* (Table S5; Figure 2).

In most cases, dung beetle assemblages sampled by pitfall traps showed lower diversity and higher dominance compared with the baseline from FITs (Table 2). We assessed this comparing the confidence intervals (CI) between FITs and pitfall traps within the same transect, when CI is not overlapping the differences are statistically significant. This is clearly observed at *Cristalino* where there is no CI overlapping, and in all transects, there are higher diversity and lower dominance in the FITs baseline assemblage than for the pitfall traps. The only exceptions were *BR‐319* transect A (pitfall traps showed greater diversity and lower dominance); transect B (where there were greater dominance in assemblages sampled by FITs); transect C (where differences were not statistically significant to Shannon index); and *Serra do Divisor* transect C (where differences were not statistically significant to Shannon index).

**TABLE 2:** Shannon and Simpson indices comparing dung beetle assemblage sampled within the same transect by flight interception traps (FITs) an pitfall traps baited with human feces at three *terra firme* primary forests within the Brazilian Amazon.

Locality	Transect	Trap type	Shannon CI 95% [L–U]	Simpson CI 95% [L–U]
*BR*‐319	A	FIT	14.369 [14.369–16.923]	8.423 [8.423–9.838]
		Pitfall	21.560 [21.560–23.356]	15.598 [15.598–16.841]
	B	FIT	13.865 [13.865–17.031]	5.999 [5.999–7.294]
		Pitfall	14.244 [14.244–15.319]	9.616 [9.616–10.270]
	C	FIT	20.364 [20.364–25.318]	11.193 [11.193–14.902]
		Pitfall	13.802 [13.802–15.111]	8.916 [8.916–9.647]
*Cristalino*	A	FIT	20.740 [20.74–22.451]	11.152 [11.152–12.321]
		Pitfall	12.470 [12.470–13.454]	6.288 [6.288–6.697]
	B	FIT	18.385 [18.385–20.576]	11.618 [11.618–12.987]
		Pitfall	5.529 [5.529–5.915]	2.678 [2.678–2.802]
	C	FIT	21.454 [21.454–27.509]	16.666 [16.666–21.054]
		Pitfall	8.797 [8.797–9.216]	5.497 [5.497–5.684]
*Serra do Divisor*	A	FIT	16.033 [16.033–22.623]	12.402 [12.402–18.794]
		Pitfall	9.563 [9.563–10.531]	4.697 [4.697–5.138]
	B	FIT	19.393 [19.393–25.148]	9.589 [9.859–12.790]
		Pitfall	14.200 [14.200–15.560]	7.145 [7.145–7.861]
	C	FIT	15.368 [15.368–20.187]	10.211 [10.211–13.636]
		Pitfall	14.580 [14.580–15.629]	8.965 [8.965–9.642]

*Note*: When confidence intervals (CI) are not overlapping the indices values are statistically different. Confidence interval with 95% [lower limit–upper limit].

## 4 DISCUSSION

Our results showed that dung beetle species are attracted to a varying extent to traps baited with human feces. We identified species that were greatly attracted (*E. hypocrita*, *O*. aff. *onorei*, *O*. aff. *rubrescens*, and *O. osculatii*) and those that were less so (*A*. aff. *frontalis*, *Co*. *degallieri*, *Co*. *telamon*, and Ca. *xanthopus*) to this type of bait (Table 1). We showed that FITs and pitfall traps result in different patterns of SRA, which we consider to be an effect of bait attractiveness, as species highly attracted to bait express a greater relative abundance in baited traps. Overall, dung beetle assemblages sampled by baited pitfall traps exhibit lower diversity and greater dominance.

The variable level of attraction to traps baited with human fecal baits can be explained by the known feeding habits of different dung beetle species. Of those species strongly attracted to detrital bait across our samples, *E. hypocrita* is known to be highly attracted to human and howler monkey (*Alouatta* spp.) feces, fish, and decaying meat (Génier, 2009). Although most *Onthophagus* species are generalist coprophages, without a clear preference for specific mammal feces (Pulido‐Herrera & Zunino, 2007), human feces is known to effectively attract *O. osculatii* (Rossini, Vaz‐de‐Mello, & Zunino, 2018) and *O*. *onorei* (Rossini, 2016). Of those species less attracted to baited traps across our samples, *Ateuchus* species are usually coprophagous, but are not necessarily attracted to human feces (Vaz‐de‐Mello et al., 1998), while *Coprophanaeus* species are preferentially necrophagous (Edmonds & Zídek, 2010). *Canthon xanthopus* is possibly a predator or specializes on decaying arthropods such as dead millipedes (Cupello & Vaz‐de‐Mello, 2018). Due to its low abundance in the FITs baseline samples (Table S3), we do not believe that Ca. *xanthopus* was attracted to decaying arthropods within the traps but was more likely to be captured during dispersal flights.

Baited pitfall traps therefore appear to disproportionally sample Scarabaeinae dung beetle species that are either coprophagous and/or generalists feeders. We confirm that noncoprophagous species or those without preference for human feces were less attracted to human feces bait in our study. This is consistent with other studies that used FIT and baited pitfall traps, which found noncoprophagous species or species with no preference for human feces (Da Silva et al., 2011). In addition, baited pitfall traps tend to capture very small counts of necrophagous (Audino et al., 2011), myrmecophilous, and termitophilous species (Ong et al., 2021), and this method is therefore likely to yield severely biased samples of dung beetle communities, which underestimate overall taxonomic and functional diversity.

Other species in our samples (*D*. aff. *batesi*, *E*. *caribaeus*, *E*. *wittmerorum*, and *S*. *proseni*) exhibited wide variation in relative abundance in baited pitfall traps depending on trap location (Table 1). *Dichotomius* species are coprophagous (F. Z. Vaz‐de‐Mello, pers. obs.); *Eurysternus caribaeus* is attracted to a wide range of feces, fish, and decaying meat; *E. wittmerorum* is mainly attracted by human feces and fish (Génier, 2009); and *S. proseni* is preferentially coprophagous and attracted to human feces (Cupello & Vaz‐de‐Mello, 2018). Since the proportional representation of these coprophagous or generalist species varies across sites, they have a lesser influence on overall dung beetle assemblage metrics. The reasons for this variation across sites are still unclear and may be related to random spatial variation and/or microclimatic factors.

Our results show that dung beetle assemblages sampled by baited pitfall traps tend to have lower diversity and higher dominance than those provided by baseline FITs (Table 2), even where species richness was higher in baited pitfall traps. We suggest that this is due to the attractiveness effect of the bait, with abundance overestimates of strongly attracted species resulting in higher relative abundance and dominance (if not hyperabundance) in our samples (Figure 2). Although this pattern was prevalent in most of our samples (Figure 2), it was not found at *BR‐319* (Table 2). However, in most cases, the assemblage structure is likely to be misrepresented due to the inherent biases introduced by the use of baits.

We emphasize that our aim focuses on the use of FITs as a baseline for community metrics and does not involve further comparisons between unbaited FITs and baited pitfall. No sampling method is completely unbiased, and both FITs and pitfall traps come with their own set of advantages and disadvantages (Table S2).

Flight interception traps represent a passive trapping technique (Matthews & Matthews, 1972) that also provides information about flight direction, an important consideration for studies of migratory insects (Henderson & Southwood, 2016) and edge effects (González et al., 2020). However, FITs may not be effective for direct population estimates of beetles as individuals in flight may successfully avoid the trap, and capture success is influenced by light intensity and wind direction (Boiteau, 2000). To optimize the chances of capture, FITs should be installed along trails or open glades (Souza et al., 2015) or along flight paths (Henderson & Southwood, 2016). FITs also represent a more costly method because they are expensive to construct or purchase (Souza et al., 2015) and require greater time investment for field installation (González et al., 2020) compared with pitfalls. FITs are also large and bulky, another potential disadvantage since this increases the likelihood of disturbance by large vertebrates (Missa et al., 2009), potentially introducing additional replacement costs.

In contrast to FITs, pitfall traps are extremely low‐cost, as cheap and widely available containers may be used for trap construction (González et al., 2020; Henderson & Southwood, 2016). They are also easy and quick to operate, ensuring robust spatial replication (Missa et al., 2009). Such considerations are especially relevant in the tropics where there is an even more pressing demand for biodiversity data and financial resources are often limited (Gardner et al., 2008). Baited pitfall traps are an efficient and economic method in terms of labour, which improves the chances of detectability of low‐densities taxa in the field (if they are attracted to the bait in use), and increases the capture success of potentially attracted species (Weinzierl et al., 2005). However, it has been suggested that pitfall traps do not provide reliable estimates of insect density (Topping & Sunderland, 1992) or relative abundance (Woodcock, 2005). Also, some preservation fluids may attract particular taxa (Greenslade & Greenslade, 1971) and their efficiency may be limited when sampling larger insects (Hancock & Legg, 2012; Spence & Niemelä, 1994).

Our results suggest that FITs provide a useful baseline for dung beetle communities, with reduced sampling biases compared with baited pitfall traps. Pitfall traps baited with fecal material clearly remain a valid, useful, and efficient tool for dung beetle surveys, providing reliable capture success for species that feed on feces. Their use in biodiversity surveys and ecological studies has been increasing over the past 30 years (Raine & Slade, 2019), many of which focus on dung beetle assemblage metrics (Bogoni et al., 2019; Chiew et al., 2021; Enari et al., 2018; Fuzessy et al., 2021; Nependa et al., 2021), and dung beetle‐mammal interaction networks (Nichols et al., 2009; Raine et al., 2018). Clearly, it is not our intention to reject a widely established and broadly accepted sampling protocol for the dung beetle field studies. Rather, we suggest that when an ecological question relies on the SRA, baited pitfall traps may often overestimate the relative abundance of species that are more strongly attracted to the bait. The main concern is that such systematic sampling bias may lead to inaccurate conclusions regarding assemblage structure. So we suggest the use of unbaited FITs to access better estimates of relative abundance of coprophagous/generalists species attracted to baits. Although tested here for dung beetles, we suggest that a similar effect could be observed for other groups of insects that are typically sampled with baited traps.

## AUTHOR CONTRIBUTIONS

**Andressa Bach:** Conceptualization (supporting); data curation (lead); formal analysis (lead); investigation (equal); methodology (equal); writing – original draft (lead). **Lúcia A. F. Mateus:** Formal analysis (supporting); methodology (equal); writing – review and editing (supporting). **Carlos A. Peres:** Conceptualization (equal); funding acquisition (equal); project administration (equal); supervision (equal); writing – review and editing (equal). **Torbjørn Haugaasen:** Conceptualization (equal); funding acquisition (equal); project administration (equal); resources (equal); writing – review and editing (equal). **Julio Louzada:** Investigation (equal); methodology (equal); writing – review and editing (equal). **Joseph E. Hawes:** Methodology (supporting); writing – review and editing (equal). **Renato A. Azevedo:** Methodology (supporting); writing – review and editing (supporting). **Emanuelly F. Lucena:** Methodology (supporting); writing – review and editing (supporting). **José Victor A. Ferreira:** Methodology (supporting). **Fernando Z. Vaz‐de‐Mello:** Conceptualization (lead); methodology (equal); supervision (lead); writing – original draft (equal).

## CONFLICT OF INTEREST STATEMENT

Authors declare no conflict of interest concerning the publication of this article.

## Supporting information


Appendix S1

## Data Availability

Data used in this study are available on Dryad. DOI: https://doi.org/10.5061/dryad.3tx95x6m5.
