# Supplementary material for: Bait attractiveness changes community metrics in dung beetles (Coleoptera: Scarabaeidae: Scarabaeinae)
Source: Ecol Evol. 2023 Apr 7;13(4):e9975. doi: 10.1002/ece3.9975 (PMC10082174; doi:10.1002/ece3.9975)
Supplement: Supplementary file 1 — Appendix S1 [file ECE3-13-e9975-s001.doc]

**Supplementary Information**

**Supplementary Figures**

Figure S1.
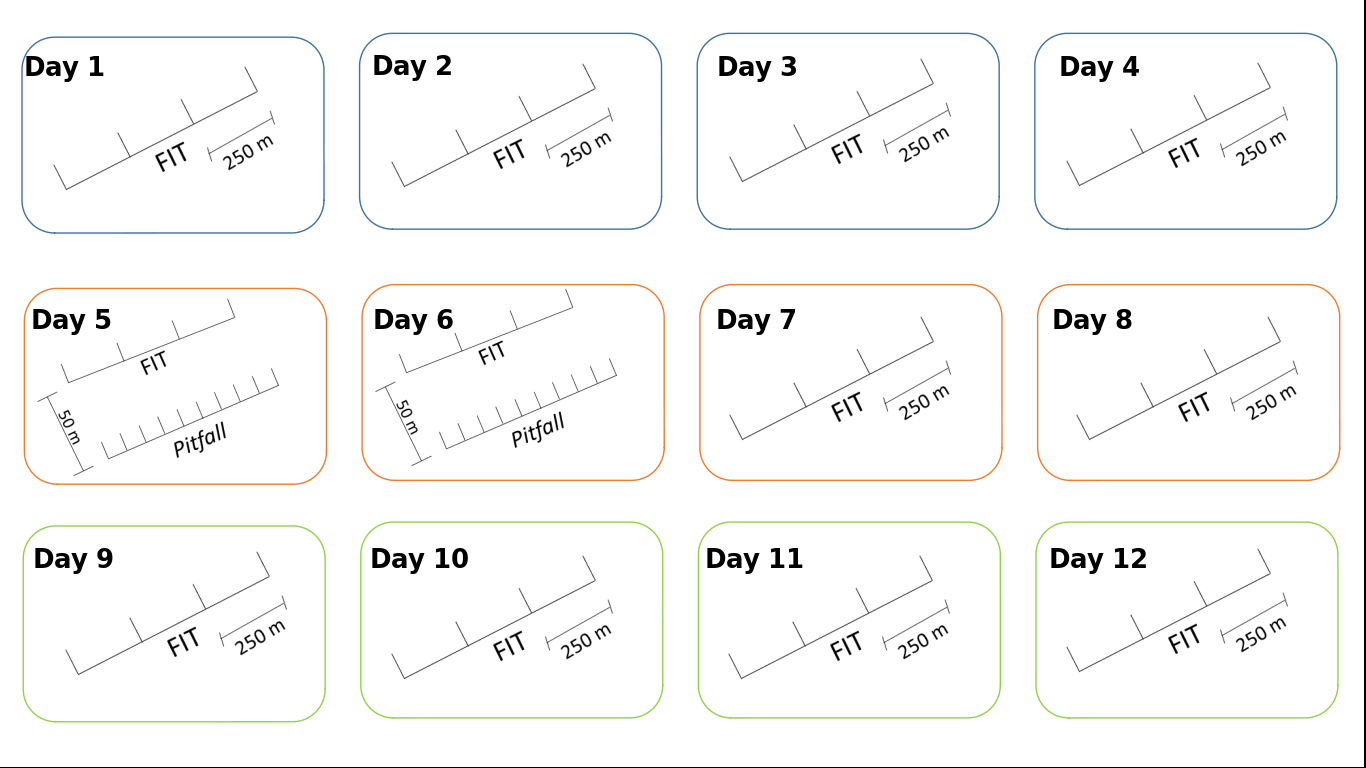
 Sampling designed used to capture dung beetles in *terra* *firme* sites of primary forest in Brazilian Amazon with flight interception traps (FIT) and baited pitfall along a transect of 1000 meters.


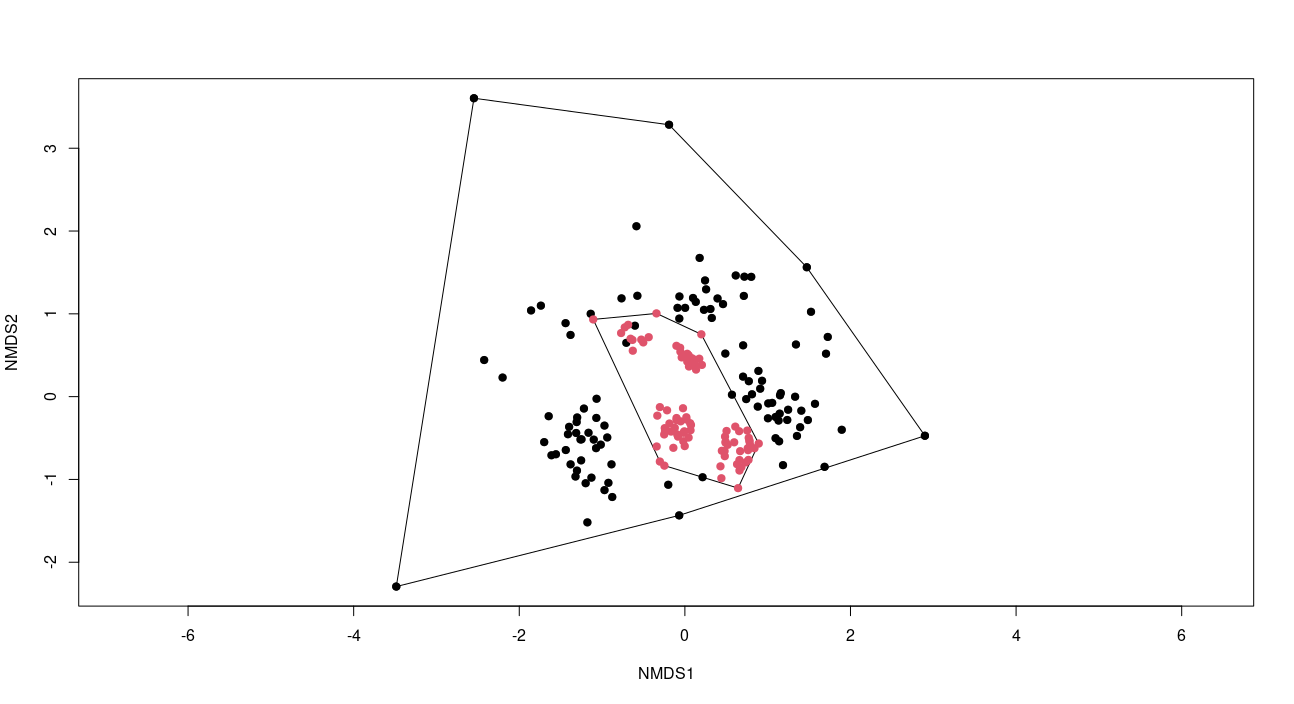
Figure S2. Non-metric multidimensional scaling of dung beetles relative abundance sampled with flight interception traps (FIT; black dots) and pitfall traps baited with human feces (pink dots) at *terra firme* sites of primary forest in Brazilian Amazon. Supporting our assumption that the expected species pool for pitfall traps is nested in the expected species pool of FIT samples.


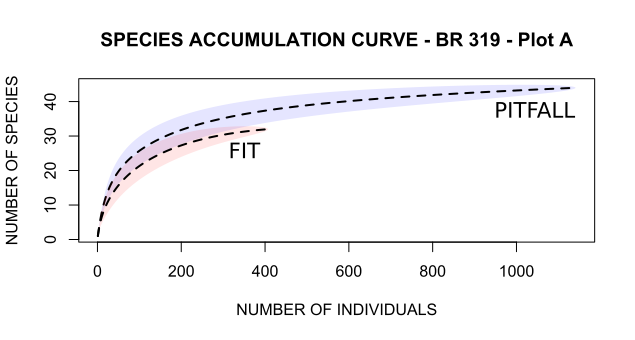

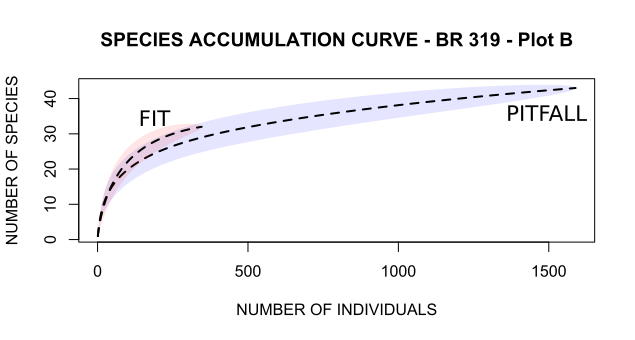

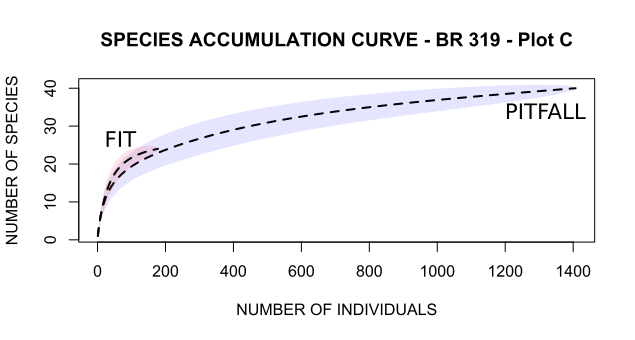

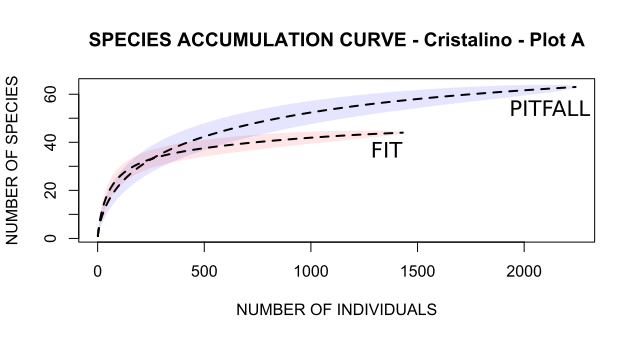

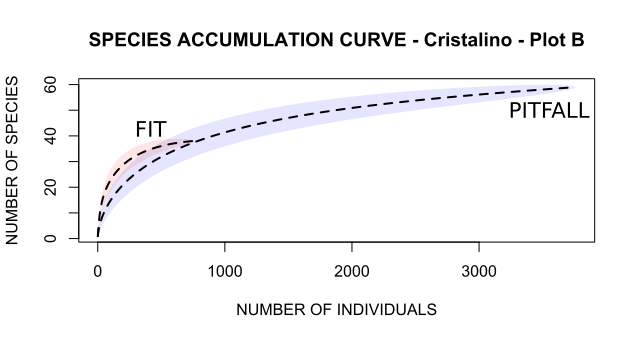

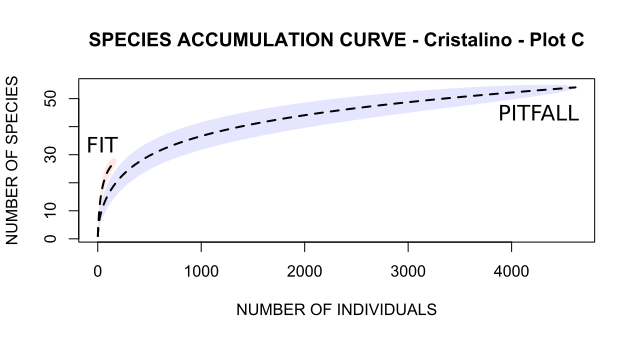


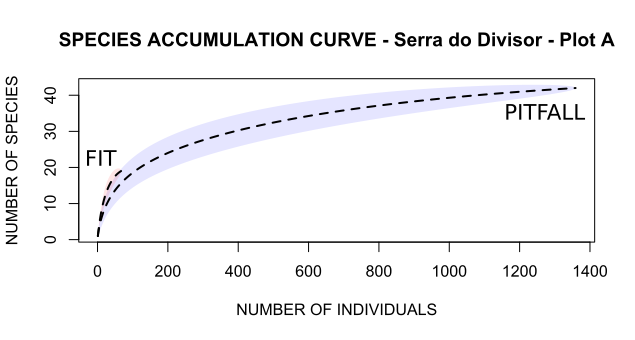

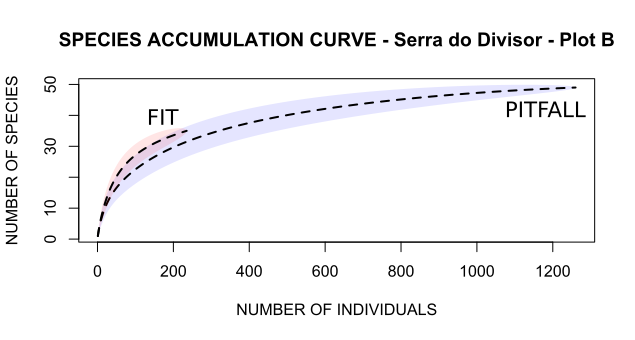

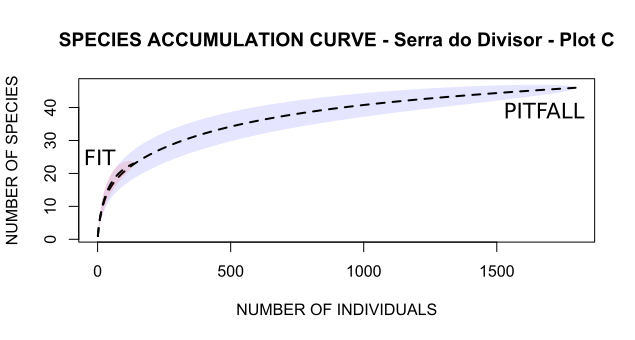


Figure S3. Species accumulation curves for dung beetles sampled at transects A, B, and C (here called plot) in *BR-*319, *Cristalino*, and *Serra do Divisor*, comparing the sampling effort (number of individuals) between samples from flight interception traps (FIT) and pitfall traps baited with human feces

**Supplementary Tables**

Table S1. Geographical coordinates for three primary forest areas of *terra firme* in the Brazilian Amazon, where dung beetle species were sampled with flight interception traps (FIT) and pitfall traps baited with human feces

| **Locality** | **Transect** | **Latitude** | **Longitude** |
| --- | --- | --- | --- |
| ***BR-319*** | A | 5°54’33.23” S | 62°23’56.49” W |
|  | B | 5°58’23.52” S | 62°27’38.26” W |
|  | C | 6°01’05.46” S | 62°29’24.87” W |
| ***Cristalino*** | A | 9°32’45.03” S | 55°54’56.74” W |
|  | B | 9°35’50.25” S | 55°53’40.08” W |
|  | C | 9°40’59.41” S | 55°54’57.60” W |
| ***Serra do Divisor*** | A | 7°25’11.4” S | 73°39'47.4" W |
|  | B | 7°28’51.0” S | 73°39'08.9" W |
|  | C | 7°26’12.0” S | 73°37'23.7" W |

Table S2. Particularities concerning captures from flight interception traps (FIT) and pitfall traps

| **Characteristics affecting capture** | **FIT** | **Pitfall traps** | **References** |
| --- | --- | --- | --- |
| **Economic cost** | FITs are expensive and more likely to be disturbed by large animals – due to their size, resulting in higher replacement costs. | Pitfall traps are low-cost, as common containers may be used. | Southwood, 1978; Souza et al., 2015; González et al., 2020. |
| **Installation time** | Approximately 15 to 30 minutes for field installation. | Quick to install, varying according to soil type. | Missa et al., 2009; González et al., 2020. |
| **Replication** | Replication is more costly because FITs are expensive. | Pitfall trap collections allow high spatial replication, as they are easy to operate and inexpensive. | Missa et al., 2009. |
| **Avoidance** | Individuals may avoid traps, because either they are able to deviate from the trap, or when they hit, they do not fall in the preservative liquid. | Individuals may avoid trap if the container edge is not buried at ground level, or escape if the inside of the container is not smooth. | Boiteau, 2000; Henderson and Southwood, 2016. |
| **Idiosyncrasy** | Capture is influenced by an individuals’ approach angle and flight height. | Pitfall trap efficiency may be limited to for larger individuals. Moreover, some taxa escape more easily. | Spence and Niemela, 1994; Boiteau, 2000; Koivula et al., 2003; Hancock and Legg, 2012; Lamarre et al., 2012; Touroult et al., 2017. |
| **Field time** | Proportionally related to capturing quality. Conversely, longer periods of exposition combined with the large size of FIT may favor disturbance by large animals. | There is no protocol for how long pitfall traps should remain in the field, varying according to the researcher question. | Ozanne, 2005; Woodcock, 2005; Missa et al., 2009; Permain et al., 2013; Souza et al., 2015. |
| **Target groups** | FIT sample active flying individuals as they move. It is efficient to capture Diptera, Coleoptera, Hymenoptera, Hemiptera, Lepidoptera, and other flying insects. | Pitfall traps capture ground-active individuals, such as Acari, millipedes, beetles, spiders, Collembola, ants, centipedes, and even crabs. | Juillet, 1963; Hosking, 1979; Peck and Davies, 1980; Lang, 2000; Lamarre et al., 2012. |

REFERENCES

Boiteau, G. (2000). Efficiency of flight interception traps for adult colorado potato beetles (Coleoptera: Chrysomelidae). *Journal of Economic Entomology*. 93(3), 630-635. https://doi.org/10.1603/0022-0493-93.3.630

González, E., Salvo, A., & Valladares, G. (2020). Insects moving through forest-crop edges: a comparison among sampling methods. *Journal of Insect Conservation*. 24(2), 249-258. http://dx.doi.org/10.1007/s10841-019-00201-6

Hancock, M.H., & Legg, C.J. (2012). Pitfall trapping bias and arthropod body mass. *Insect Conservation and Diversity*. 5(4), 312-318. https://doi.org/10.1111/j.1752-4598.2011.00162.x

Henderson, P.A., & Southwood, T.R.E. (2016). Ecological Methods, (4th ed).

Hosking G.P. (1979). Trap comparison in the capture of flying Coleoptera. *New Zealand Entomologist*. 7(1), 87–92. https://doi.org/10.1080/00779962.1979.9722338

Juillet, J.A. (1963). A comparison of four types of traps used for capturing flying insects. *Canadian Journal of Zoology*. 41(2), 219-223. https://doi.org/10.1139/z63-023

Koivula, M., Kotze, D.J., Hiisivuosi, L. & Rita, H. (2003) Pitfall trap efficiency: do trap size, collecting fluid and vegetation structure matter? *Entomologica Fennica*. 14, 1-14. https://doi.org/10.33338/ef.84167

Lang, A. (2000). The pitfalls of pitfalls: a comparison of pitfall trap catches and absolute density estimates of epigeal invertebrate predators in arable land. *Anzeiger für Schädlingskunde*. 73(4), 99-106. https://doi.org/10.1007/BF02956438

Lamarre, G.P.A., Molto, Q., Fine, P.V.A., & Baraloto, C. (2012). A comparison of two common flight interception traps to survey tropical arthropods. *Zookeys*. 216, 43-55. https://doi.org/10.3897//zookeys.216.3332

Missa, O., Basset, Y., Alonso, A., Miller, S.E., Curletti, G., Meyer, M., Eardley, C., Mansell, M.W., & Wagner, T. (2009). Monitoring arthropods in a tropical landscape: relative effects of sampling methods and habitat types on trap catches. *Journal of Insect Conservation*. 13(1), 103-118. https://doi.org/10.1007/s10841-007-9130-5

Ozanne, C.M.P. (2005). Sampling methods for forest understory vegetation. In: Leather, S.R. (Ed.), *Insect Sampling in Forest Ecosystems* (pp.58-76).

Permain, G.; Dufrêne, M.; Brin, A. & Bouget, C. (2013). Influence of sampling effort on saproxylic beetle diversity assessment: implications for insect monitoring studies in European temperate forests. *Agricultural and Forest Entomology*. 15, 135-145. https://doi.org/10.1111/afe.12008

Southwood, T.R.E. (1978). Ecological Methods With Particular Reference to the Study of Insect Populations (2nd ed.).

Souza, M.M, Perillo, L.N., Barbosa, B.C., & Prezoto, F. (2015). Use of flight interception traps of Malaise type and attractive traps for social wasps record (Vespidae: Polistinae). *Sociobiology*. 62(3), 450-456. https://doi.org/10.13102/sociobiology.v62i3.708

Spence, J.R, & Niemelä, J.K. (1994). Sampling carabid assemblages with pitfall traps: the madness and the method. *The Canadian Entomologist*. 126(3), 881-894. https://doi.org/10.4039/Ent126881-3

Touroult, J., Dalens, P.H., Giuglaris, J.L., Lapèze, J., & Boilly, O. (2017). Structure des communautés de Phanaeini (Coleoptera: Scarabaeidae) de Guyane: Étude par échantillonnage massif au piège d’interception. *Annales de la Société entomologique de France* (NS). 53(3), 143-161. https://doi.org/10.1080/00379271.2017.1319294

Woodcock, B.A. (2005). Pitfall trapping in ecological studies. In: Leather, S.R. (Ed.) *Insect Sampling in Forest Ecosystems* (pp. 37-57).

Table S3. List of dung beetle (Coleoptera: Scarabaeidae: Scarabaeinae) species sampled at three *terra firme* primary forest in the Brazilian Amazon (*BR*-319, *Cristalino*, and *Serra do Divisor*) with flight interception traps and pitfall traps baited with human feces. There is a footnote attached to the table with information about the species and the data analysis

| **Species** | | | |
| --- | --- | --- | --- |
| *Anomiopus batesi* | *Canthidium* sp06 | *Coprophanaeus* sp01 | *Eurysternus harlequin^d^* |
| *Anomiopus* sp01 | *Canthidium* sp07 | *Coprophanaeus lancifer^d^* | *Eurysternus hypocrita*^b4c1c3d^ |
| *Anomiopus* sp02^a^ | *Canthidium* sp08 | *Coprophanaeus telamon*^b4c1c2c3d^ | *Eurysternus jessopi^d^* |
| *Anomiopus* sp03^a^ | *Canthidium* sp09^d^ | *Coprophanaeus terrali^a^* | *Eurysternus plebejus^d^* |
| Anomiopus sp04 | *Canthidium* sp10^a^ | *Cryptocanthon campbellorum^d^* | *Eurysternus strigilatus*^b2c1c3d^ |
| *Anomiopus* sp05 | *Canthidium* sp11 | *Cryptocanthon peckorum*^b2c3d^ | *Eurysternus uniformis^d^* |
| *Anomiopus* sp06^a^ | *Canthidium* sp12^d^ | *Deltochilum* sp01^d^ | *Eurysternus vastiorum^ad^* |
| *Anomiopus* sp07^a^ | *Canthidium* sp13^d^ | *Deltochilum* sp02^d^ | *Eurysternus ventricosus*^b3d^ |
| *Anomiopus* sp08^a^ | *Canthidium* sp14 | *Deltochilum* sp03^a^ | *Eurysternus wittmerorum*^b4c1c2c3d^ |
| *Anomiopus* sp09 | *Canthidium* sp15^a^ | *Deltochilum* sp04^d^ | *Eutrichillum* sp01 |
| *Anomiopus* sp10^a^ | *Canthidium* sp16^d^ | *Deltochilum* sp05^d^ | *Isocopris* sp01^d^ |
| *Anomiopus pictus^a^* | *Canthidium* sp17^d^ | *Deltochilum carinatum*^b3c2d^ | *Isocopris imitator^d^* |
| *Ateuchus* aff. *aeneomicans*^b3d^ | *Canthidium* sp18 | *Deltochilum* sp06^ad^ | *Isocopris nitidus^d^* |
| *Ateuchus* aff. *freudei^d^* | *Canthidium* sp19^d^ | *Deltochilum* sp07 | *Ontherus* aff. *alexis^d^* |
| *Ateuchus* aff. *frontalis*^b3d^ | *Canthidium* sp20^d^ | *Deltochilum* sp08^d^ | *Ontherus* aff. *edentulus^a^* |
| *Ateuchus* aff. *murrayi^d^* | *Canthidium* sp21^d^ | *Deltochilum* sp09^d^ | *Ontherus raptor^a^* |
| *Ateuchus* aff. *pygidialis* sp01^d^ | *Canthidium* sp22^d^ | *Deltochilum* sp10^d^ | *Onthophagus* aff. *onorei*^b1c1c2d^ |
| *Ateuchus* aff. *pygidialis* sp02^d^ | *Canthidium* sp23 | *Deltochilum* sp11 | *Onthophagus* aff. *osculatii*^b1c1d^ |
| *Ateuchus* sp01^d^ | *Canthidium* sp24^d^ | *Deltochilum* sp12^d^ | *Onthophagus* aff. *rubrescens*^b1c1c2d^ |
| *Ateuchus* sp02^d^ | *Canthidium* sp25 | *Deltochilum enceladus^d^* | *Onthophagus* sp01 |
| *Ateuchus* sp03^d^ | *Canthidium* sp26 | *Deltochilum orbiculare*^b4c2c3d^ | *Onthophagus* sp02^d^ |
| *Ateuchus* sp04^d^ | *Canthidium* sp27^a^ | *Deltochilum orbignyi amazonicum*^b4c1c3d^ | *Onthophagus digitifer*^b3d^ |
| *Ateuchus* sp05^d^ | *Canthidium* sp28^d^ | *Deltochilum schefflerorum*^b3^ | *Onthophagus onorei*^b3d^ |
| *Ateuchus* sp06^d^ | *Canthidium* sp29^d^ | *Deltorhinum* aff. *vazdemelloi* | *Onthophagus onthochromus^d^* |
| Ateuchus sp07ad | *Canthidium* sp30^d^ | *Dendropaemon angustipennis*^b1^ | *Onthophagus osculatii*^b3c2c3d^ |
| *Ateuchus* sp08^d^ | *Canthidium* sp31^a^ | *Dendropaemon lydiae^a^* | *Onthophagus rubrescens^d^* |
| *Ateuchus* sp09^d^ | *Canthidium* sp32^ad^ | *Dichotomius* aff. *batesi*^b4c1c3d^ | *Onthophagus xanthomerus^d^* |
| *Ateuchus* sp10 | *Canthidium* *stofeli*^b1^ | *Dichotomius* aff. *conicollis* | *Oxysternon conspicillatum*^b3c2c3d^ |
| *Ateuchus* sp11^d^ | *Canthon* aff. *angustatus^d^* | *Dichotomius* aff. *lucasi*^b2c1d^ | *Oxysternon macleayi^d^* |
| *Ateuchus* sp12 | *Canthon bimaculatus^d^* | *Dichotomius* aff. *lucasi* sp01^d^ | *Oxysternon silenus*^b3c3d^ |
| *Ateuchus substriatus*^b4c1c2d^ | *Canthon* sp01 | *Dichotomius* aff. *lucasi* sp02^d^ | *Oxysternon spiniferum curvispinum^d^* |
| *Bdelyrus* sp01^a^ | *Canthon brunneus^d^* | *Dichotomius* aff. *podalirius^d^* | *Phanaeus bispinus^b2d^* |
| *Bdelyrus* sp02^a^ | *Canthon conformis^ad^* | *Dichotomius* aff. *pseudocuprinus^d^* | *Phanaeus cambeforti^b2c1c3d^* |
| *Bdelyrus paraensis^a^* | *Canthon* sp02 | *Dichotomius apicalis^d^* | *Phanaeus chalcomelas^b4c1c2c3d^* |
| *Besourenga horacioi^d^* | *Canthon* sp03^d^ | *Dichotomius carinatus^d^* | *Phanaeus sp01^d^* |
| *Canthidium* aff. *cupreum^d^* | *Canthon* sp04^a^ | *Dichotomius gandinii*^b2d^ | *Phanaeus sororibispinus* |
| *Canthidium* aff. *dohrni^d^* | *Canthon fulgidus fulgidus^d^* | *Dichotomius mamillatus*^b4c2d^ | *Scybalocanthon aereusa^d^* |
| *Canthidium* aff. *funebre*^b3d^ | *Canthon fulgidus pererai^d^* | *Dichotomius melzeri*^b1d^ | *Scybalocanthon uniplagiatus^b2c1d^* |
| *Canthidium* aff. *gerstaeckeri^d^* | *Canthon luteicollis*^b2c1d^ | *Dichotomius nimuendaju*^b1d^ | *Sulcophanaeus faunus^d^* |
| *Canthidium* aff. *lentum^d^* | *Canthon nitidicolis^d^* | *Dichotomius ohausi^d^* | *Sylvicanthon attenboroughi^b1c1d^* |
| *Canthidium* aff. *melanacephalum^d^* | *Canthon quadrimaculatus^da^* | *Dichotomius podalirius^d^* | *Sylvicanthon bridarollii^d^* |
| *Canthidium* aff. *onitoides*^b2c1d^ | *Canthon* aff. *histrio^d^* | *Dichotomius robustus*^b4d^ | *Sylvicanthon proseni^b4c1c2d^* |
| *Canthidium* aff. *onthophagoides* | *Canthon rufocoeruleus*^b3d^ | *Dichotomius worontzowi^d^* | *Trichillum sp01^d^* |
| *Canthidium* aff. *orbiculatum^d^* | *Canthon semiopacus*^b2d^ | *Eurysternus arnaudi*^b1c1c2d^ | *Uroxys sp01^d^* |
| *Canthidium* aff. *rufinum^d^* | *Canthon xanthopus*^b2c1d^ | *Eurysternus atrosericus^d^* | *Uroxys sp02^ad^* |
| *Canthidium* sp01^d^ | *Canthonella* sp01^d^ | *Eurysternus caribaeus*^b4c1c2c3d^ | *Uroxys sp03^d^* |
| *Canthidium* sp02^d^ | *Canthonella* sp02 | *Eurysternus cayennensis*^b2c1d^ | *Uroxys sp04^d^* |
| *Canthidium* sp03 | *Canthonella* sp03 | *Eurysternus cyclops^d^* | *Uroxys sp05^a^* |
| *Canthidium* sp04^d^ | *Canthonella* sp04^ad^ | *Eurysternus foedus*^b4d^ |  |
| *Canthidium* sp05^d^ | *Coprophanaeus degallieri*^b1d^ | *Eurysternus hamaticollis*^b3d^ |  |

^a^Singletons excluded for Hotelling’s T² test. ^b^Species considered to IndVal, ^b1^Species common to BR319 and *Cristalino*, ^b2^Species common to *BR-319* and *Serra do Divisor* Park, ^b3^Species common to *Cristalino* and *Serra do Divisor*, ^b4^Species sampled at all sites. ^c^Species used in Chi-Squared Goodness of Fit, ^c1^*BR-319*, ^c2^*Cristalino*, ^c3^*Serra do Divisor*. ^d^Species captured by pitfall traps.

Table S4. Chi-square Goodness of Fit results by locality, used to compared species’ proportional abundance for dung beetles sampled by flight interception traps (FIT) and pitfall traps baited with human feces within the same transect at three primary forest areas of *terra firme* in the Brazilian Amazon

| **Locality** | **Transect** | **χ²** | **DF** | **p** |
| --- | --- | --- | --- | --- |
| ***BR-319*** | A | 5,145.9 | 18 | <0.0001 |
|  | B | 3,498.8 | 16 | <0.0001 |
|  | C | 3,630.2 | 14 | <0.0001 |
| ***Cristalino*** | A | 8,394.6 | 18 | <0.0001 |
|  | B | 10.865 | 17 | <0.0001 |
|  | C | 16.636 | 13 | <0.0001 |
| ***Serra do Divisor*** | A | 374.82 | 10 | <0.0001 |
|  | B | 3,491.3 | 22 | <0.0001 |
|  | C | 9,576.3 | 13 | <0.0001 |

Table S5. Standardized residuals (SR) of Chi-Square Goodness for dung beetles sampled by flight interception traps (FIT) and pitfall traps baited with human feces within the same plot at three primary forest areas of *terra firme* in the Brazilian Amazon (*BR-319*, *Cristalino*, and *Serra do Divisor*). Species that contributed to significance were those with SR outside of the range -1.96 to 1.96. When SR<-1.96, species showed a lower proportional abundance in pitfall trap samples than in FIT; when SR>1.96, species showed a greater proportional abundance in pitfall traps samples than in FIT

| **Species** | **Locality** | **Transect** | **SR** |
| --- | --- | --- | --- |
| *Ateuchus* aff. *aeneomicans* | *Serra do Divisor* | B | 1.7¹ |
| *Ateuchus* aff. *frontalis*^a^ | *Cristalino* | A | -13.35 |
|  |  | B | -29.08 |
| *Ateuchus substriatus* | *BR-319* | A | -1.71¹ |
|  |  | B | -13.31 |
|  |  | C | -6.67 |
|  | *Cristalino* | A | -5.11 |
|  |  | C | -8.11 |
| *Canthidium* aff. *onitoides* | *BR-319* | A | -0.75¹ |
| *Canthon luteicollis* | *BR-319* | A | 4.97 |
|  | *Serra do Divisor* | B | 15.74 |
| *Canthon xanthopus*^a^ | *BR-319* | C | -4.95 |
| *Coprophanaeus degallieri*^a^ | *Cristalino* | B | -10.39 |
| *Coprophanaeus telamon*^a^ | *BR-319* | A | -4.3 |
|  |  | B | -9.03 |
|  |  | C | -6.39 |
|  | *Cristalino* | A | -6.15 |
|  |  | B | -13.37 |
|  |  | C | -24.03 |
|  | *Serra do Divisor* | B | -4.88 |
|  |  | C | -11.71 |
| *Cryptocanthon peckorum* | *Serra do Divisor* | A | -4.47 |
|  |  | B | -2.76 |
|  |  | C | -2.17 |
| *Deltochilum carinatum* | *Cristalino* | A | -5.11 |
|  |  | B | -8.59 |
|  |  | C | -5.79 |
|  | *Serra do Divisor* | B | -2.006 |
| *Deltochilum orbiculare* | *Cristalino* | A | -12.02 |
|  |  | B | -11.63 |
|  |  | C | -23.18 |
|  | *Serra do Divisor* | B | -1.86¹ |
|  |  | C | -0.21¹ |
| *Deltochilum orbignyi amazonicum* | *BR-319* | A | 0.65¹ |
|  |  | B | -9.48 |
|  |  | C | -4.35 |
|  | *Cristalino* | A | -2.61 |
|  |  | B | -4.08 |
|  | *Serra do Divisor* | A | -1.63¹ |
|  |  | B | -0.95¹ |
|  |  | C | -3.82 |
| *Dichotomius* aff. *batesi*^b^ | *BR-319* | B | 4.13 |
|  |  | C | 3.95 |
|  | *Cristalino* | A | 6.47 |
|  |  | B | -7.99 |
|  | *Serra do Divisor* | B | -0.978¹ |
|  |  | C | 1.48¹ |
| *Dichotomius* aff. *lucasi* | *BR-319* | A | -5.17 |
|  |  | B | -6.86 |
|  |  | C | -7.87 |
|  | *Serra do Divisor* | A | -11.17 |
| *Dichotomius mamillatus* | *Cristalino* | A | -2.61 |
|  |  | B | -5.68 |
|  |  | C | -12.25 |
|  | *Serra do Divisor* | B | -1.41¹ |
| *Dichotomius melzeri* | *Cristalino* | A | 0.033¹ |
| *Eurysternus arnaudi* | *BR-319* | A | 1.19¹ |
|  |  | B | -2.87 |
|  |  | C | -2.9 |
|  | *Cristalino* | A | -3.09 |
|  |  | C | -8.74 |
| *Eurysternus caribaeus*^b^ | *BR-319* | A | 11.01 |
|  |  | B | 18.19 |
|  |  | C | 5.96 |
|  | *Cristalino* | A | -4.87 |
|  |  | B | -19.57 |
|  |  | C | -22.6 |
|  | *Serra do Divisor* | A | -6.02 |
|  |  | B | 11.04 |
|  |  | C | -4.31 |
| *Eurysternus cayennensis* | *BR-319* | A | -15.12 |
|  |  | B | -17.9 |
|  |  | C | -8.74 |
| *Eurysternus cayennensis* | *Serra do Divisor* | A | 11.24 |
|  |  | B | -0.35¹ |
| *Eurysternus foedus* | *Serra do Divisor* | B | -0.37¹ |
| *Eurysternus hamaticollis* | *Cristalino* | B | -4.13 |
| *Eurysternus hypocrita*^b^ | *BR-319* | C | 6.19 |
|  | *Cristalino* | A | 3.56 |
|  |  | B | 1.47¹ |
|  | *Serra do Divisor* | B | 13.52 |
|  |  | C | 7.55 |
| *Eurysternus strigilatus* | *BR-319* | A | 0.65¹ |
|  |  | B | -4.52 |
|  | *Serra do Divisor* | A | -3.45 |
|  |  | B | -6.81 |
|  |  | C | -5.49 |
| *Eurysternus wittmerorum*^b^ | *BR-319* | A | 21.16 |
|  |  | B | 21.55 |
|  | *Cristalino* | A | -9.3 |
|  |  | B | -12.86 |
|  |  | C | -4.6 |
|  | *Serra do Divisor* | B | 22.7 |
|  |  | C | 18.6 |
| *Onthophagus* aff. *onorei*^b^ | *BR-319* | A | 35.07 |
|  |  | B | 22.59 |
|  |  | C | 33.48 |
|  | *Cristalino* | A | 4.09 |
|  |  | B | 10.81 |
|  |  | C | 100.77 |
| *Onthophagus* aff. *osculatii* | *BR-319* | A | 7.39 |
|  |  | B | 20.04 |
|  |  | C | 2.12 |
| *Onthophagus* aff. *rubrescens*^b^ | *BR-319* | A | 16.88 |
|  |  | B | 23.6 |
|  |  | C | 45.43 |
|  | *Cristalino* | A | 86.42 |
|  |  | B | 97.25 |
|  |  | C | 58.54 |
| *Onthophagus osculatii*^b^ | *Cristalino* | A | 23.18 |
|  |  | B | 23.43 |
|  |  | C | 38.23 |
|  | *Serra do Divisor* | A | 10.28 |
|  |  | B | 41.74 |
|  |  | C | 94.75 |
| *Oxysternon conspicillatum* | *Cristalino* | A | -2.49 |
|  |  | B | -5.16 |
|  |  | C | -9.74 |
| *Oxysternon conspicillatum* | *Serra do Divisor* | A | -1.07¹ |
|  |  | B | -2.44 |
|  |  | C | -0.58¹ |
| *Oxysternon silenus* | *Serra do Divisor* | B | -3.69 |
|  |  | C | -5.79 |
| *Phanaeus bispinus* | *Serra do Divisor* | B | -1.41¹ |
| *Phanaeus cambeforti* | *BR-319* | A | -1.9¹ |
|  |  | B | -4.73 |
|  | *Serra do Divisor* | A | -0.88¹ |
|  |  | B | -4.68 |
|  |  | C | -4.61 |
| *Phanaeus chalcomelas* | *BR-319* | A | -8.45 |
|  |  | B | -7.53 |
|  |  | C | -12.44 |
|  | *Cristalino* | A | 0.033¹ |
|  |  | B | -7.6 |
|  |  | C | -22.35 |
|  | *Serra do Divisor* | B | -1.92¹ |
|  |  | C | -8.23 |
| *Scybalocanthon uniplagiatus* | *BR-319* | A | -12.07 |
|  |  | B | -8.13 |
|  |  | C | -10.25 |
|  | *Serra do Divisor* | A | -3.04 |
|  |  | B | -1.12 |
| *Sylvicanthon attenboroughi* | *BR-319* | A | -2.1 |
| *Sylvicanthon proseni*^b^ | *BR-319* | A | 51.55 |
|  |  | B | 23.69 |
|  | *Cristalino* | A | -3.1 |
|  |  | B | -6.35 |
|  |  | C | -2.74 |
| *Sylvicanthon proseni*^b^ | *Serra do Divisor* | A | -2.91 |
|  |  | B | 25.15 |
| ¹Not statistically significant. ^a^Species weakly associated with FIT. ^b^Species highly associated with pitfall | | | |

Table S6. The Point-Biserial Correlation Coefficient of species highly, moderately, and weakly associated with flight interception traps (FIT) and pitfall traps baited with human feces, according to IndVal results regarding dung beetle species sampled with FIT and baited pitfall at three primary forest areas of *terra firme* in the Brazilian Amazon

| **Association Strength** | **Species** | **Point-Biserial Correlation Coefficient to pitfall traps** | **p** |
| --- | --- | --- | --- |
| **Weakly associated with FIT** | *Ateuchus* aff. *frontalis* | -0.289 | 0.001 |
|  | *Coprophanaeus telamon* | -0.171 | 0.016 |
|  | *Coprophanaeus degallieri* | -0.264 | 0.004 |
|  | *Canthon xanthopus* | -0.193 | 0.011 |
|  | *Dendropaemon angustipennis* | -0.243 | 0.006 |
| **Highly associated with pitfall** | *Onthophagus* *aff.* *rubrescens* | 0.479 | 0.001 |
|  | *Onthophagus osculatii* | 0.692 | 0.001 |
|  | *Onthophagus* *aff. onorei* | 0.434 | 0.001 |
|  | *Eurysternus caribaeus* | 0.529 | 0.001 |
|  | *Eurysternus hypocrita* | 0.514 | 0.001 |
|  | *Eurysternus wittmerorum* | 0.364 | 0.001 |
|  | *Dichotomius* *aff. batesi* | 0.384 | 0.001 |
|  | *Sylvicanthon proseni* | 0.495 | 0.001 |
| **Moderately associated with pitfall** | *Canthon luteicollis* | 0.443 | 0.001 |
|  | *Onthophagus onorei* | 0.694 | 0.001 |
|  | *Onthophagus* aff. *osculatii* | 0.334 | 0.001 |
|  | *Eurysternus cayennensis* | 0.351 | 0.001 |
| **Weakly associated with pitfall** | *Eurysternus arnaudi* | 0.311 | 0.001 |
|  | *Oxysternon conspicillatum* | 0.268 | 0.004 |
|  | *Eurysternus hamaticollis* | 0.449 | 0.001 |
|  | *Eurysternus strigilatus* | 0.146 | 0.085^a^ |
|  | *Deltochilum orbiculare* | 0.113 | 0.105^a^ |
|  | *Dichotomius mamillatus* | 0.23 | 0.002 |
|  | *Eurysternus foedus* | 0.316 | 0.001 |
|  | *Eurysternus ventricosus* | 0.319 | 0.001 |
|  | *Canthon rufocoeruleus* | 0.249 | 0.005 |
|  | *Dichotomius robustus* | 0.219 | 0.003 |
|  | *Onthophagus digitifer* | 0.229 | 0.013 |
|  | *Dichotomius melzeri* | 0.163 | 0.056^a^ |
|  | *Canthidium* aff. *funebre* | 0.209 | 0.016 |

^a^Species without significant correlation.
